# Healthcare overview: new perspectives

**DOI:** 10.1186/1878-5085-5-S1-A13

**Published:** 2014-02-11

**Authors:** Vincenzo Costigliola

**Affiliations:** 1 Book Editor, The European Association for Predictive, Preventive and Personalised Medicine, Brussels, Belgium

## 

In healthcare, the realisation of an optimistic prognosis against pessimistic ones depends on current innovations in diagnostic and cost-effective treatment approaches being widely adopted in clinical practice. Utilisation of advanced early and predictive diagnostics, targeted prevention and personalised medical approaches could enable the elderly subpopulation to reach the 100-year age limit in good physical and mental health, as actively contributing members of society. This task requires intelligent political regulations and creation of new guidelines to advance current healthcare systems.

**Figure 1:**
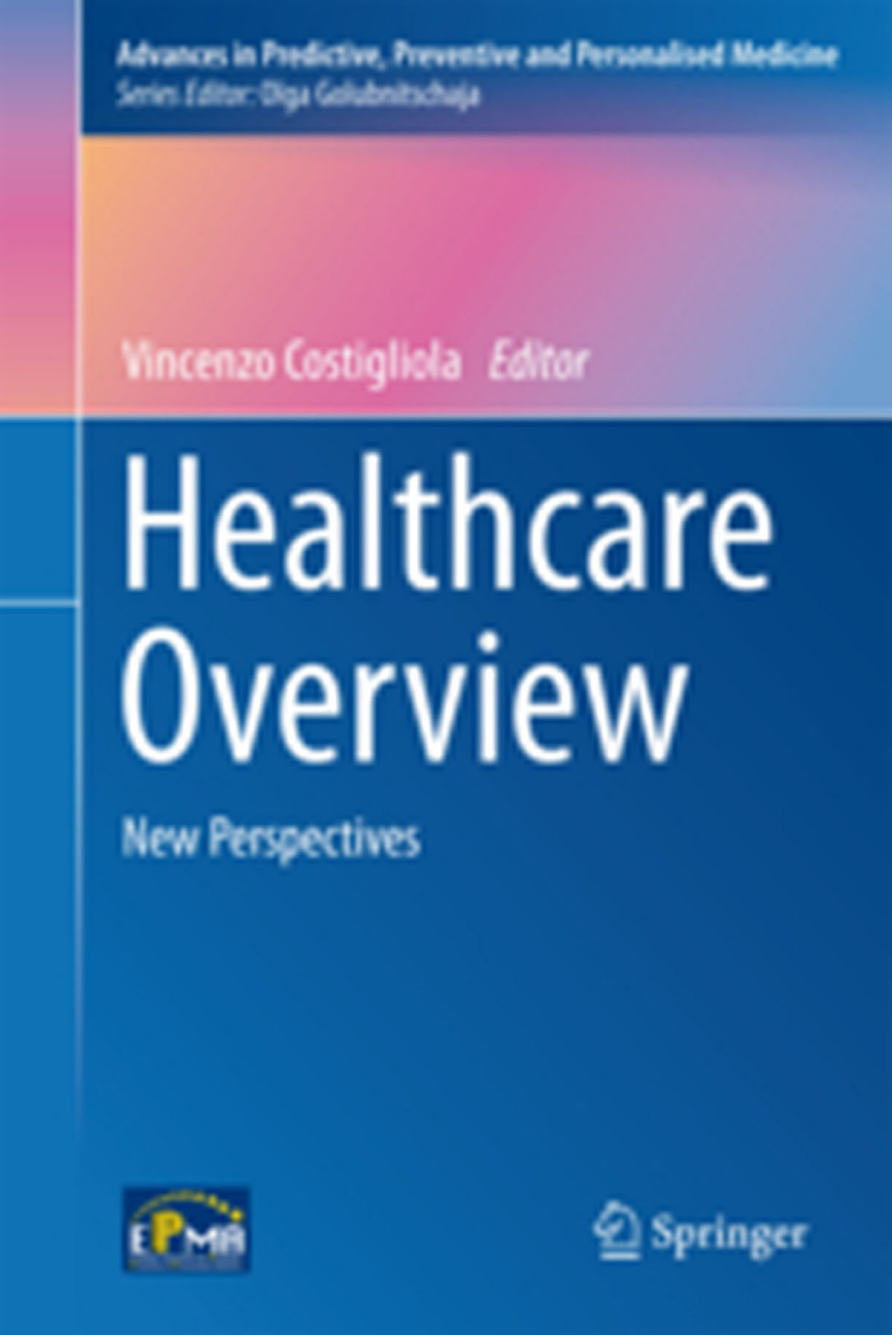
Book Volume ISBN 978-94-007-4602-2 in the EPMA / Springer Book Series “Advances in Predictive, Preventive and Personalised Medicine, http://www.springer.com/biomed/book/978-94-007-4601-5

The European Association for Predictive, Preventive and Personalised Medicine (EPMA) has created a robust platform to discuss the topic which the current book volume (Figure 1) is dedicated to, namely, an overview of healthcare and professional outlook for its specific branches. On a global scale, this is a unique concept presenting

■ comprehensive review of historic, cultural, demographic, ethnic, socio-economical, political, and other aspects which contribute to realisation of current healthcare systems;

■ comparisons of data / information both from Europe and worldwide in order to share issue-related experiences and to learn from each other about advantages and disadvantages of single healthcare systems.

In this book-volume, we have collected contributions from 15 countries that cover geopolitical regions in around the globe. The contributions provide expert opinions on timely and highly-relevant topics:

➣ Personalisation of medical approaches in global scale

➣ Integrative view and multimodal approaches to treat the patient individually and effectively

➣ Overview of individual healthcare systems in the global context

➣ Overview of current healthcare-responsible institutions and stakeholders

➣ Innovative national and international programmes dedicated to predictive and preventive medicine

➣ Current position of the pharmaceutical industry in PPPM

➣ Systematic approaches of well-being concepts

➣ Crucial aspects of individualised nutrition to enable PPPM

➣ Traditional and non-conventional medicine as one of the cost-effective treatment approaches towards healing beyond surgery and medication

➣ Gender particularities to be respected in PPPM

➣ Health promotion and work

➣ The role of laboratory medicine in healthcare

➣ Standards and management in PPPM

➣ Economy of predictive, preventive and personalised medicine

➣ Ethical paradigm shift in PPPM

➣ Future outlook - new perspectives in healthcare in the context of predictive, preventive and personalised medicine.

The main objectives of these efforts are to mark the stakeholders in the field, to consolidate professional groups and to prepare expert recommendations of how to optimise approaches for cost-effective healthcare focused on the patient.

